# Barriers and facilitators to scale-up of hospital-at-home: an observational cohort study protocol

**DOI:** 10.3389/frhs.2025.1571090

**Published:** 2025-06-06

**Authors:** Stephanie Q. Ko, Shi Yun Low, Nick Sevdalis

**Affiliations:** ^1^ Department of Medicine, National University Hospital, Singapore, Singapore; ^2^ Behavioural and Implementation Science Interventions (BISI), National University of Singapore, Singapore, Singapore; ^3^ NUHS@Home, National University Health System (Singapore), Singapore, Singapore

**Keywords:** Hospital-at-Home, Singapore, barriers and facilitators, EPIS framework, scale-up framework, contextual influences

## Abstract

**Introduction:**

Hospital-at-Home interventions have been shown to be clinically and cost-effective, and many healthcare systems internationally are investing in scaling-up such interventions. However, most existing studies focus on how effective the intervention is, rather than how to successfully scale it up. We report a study protocol for a theory-driven investigation of a Hospital-at-Home intervention. We propose a novel combination of two established implementation science frameworks—the EPIS framework and the Scale-Up framework—and apply it to a planned scale-up of a Hospital-at-Home intervention in Singapore.

**Methods:**

and analysis: This will be an observational cohort study across 23 months (May 2022 to April 2024) to evaluate the association of outer and inner contextual factors on key implementation outcomes—the volume of patients admitted, operational efficiency and levels of adoption. Statistical process control graphs will be used to examine variation in the implementation outcomes over time. Linear regression will be applied to assess associations of outcomes with contextual factors that are continuous variables; logistic regression will be applied to assess the associations of outcomes with binary/descriptive contextual factors. To supplement these, qualitative methods will be applied using a content analysis of monthly meeting minutes and focus group discussions with the implementation team to understand and explain the outcomes of the observational cohort study.

**Ethics and dissemination:**

This protocol has been reviewed and approved by the National Health Group Domain Specific Review Board: Reference Number: 2023/00245. Apart from the end-of-study focus group discussions, waiver of informed consent was sought as the data sources were a review of routinely collected retrospective data. The results of this study will be disseminated to peer-reviewed journals, presented at conferences and shared with policy-level stakeholders.

## Introduction

In many health systems, the demand for hospital beds driven by ageing populations is increasing faster than the available supply. As a result, sustainable and effective alternatives to hospitalization are becoming increasingly important. One such strategy is the Hospital-at-Home (HaH) service, which substitutes traditional inpatient care with treatment delivered in the patient's home (1). HaH is a complex intervention providing hospital-level services at home that involves a daily “ward round” via virtual or home visits, administration of intravenous therapy, and simple investigations at home, with 24/7 access to the care team. These services can be technology-enabled, utilizing remote monitoring and telecommunication systems. Across multiple randomized controlled trials conducted in Australia, Europe, and the USA, HaH interventions have demonstrated similar or improved clinical outcomes and positive patient experiences, often at a lower cost to the healthcare system (2–5).

In recent years, many healthcare systems have invested heavily in the development of HaH programs, including Singapore (Mobile Inpatient Care @ Home) (6), NHS England (Hospital-at-Home/Virtual Wards) (7, 8), and the Center for Medicaid and Medicare Services (Hospital-at-Home) in the United States (9). Although a number of studies have addressed implementation (10, 11), most ongoing evaluations remain focused on safety, efficacy, and cost-effectiveness (2, 3). Despite generally positive results, many pilot programs struggle to scale from a census of 3–5 patients per day to 50–100 patients. Apart from limitations in operational models, we believe that a limited understanding of implementation factors and the lack of systematic application of implementation science concepts (as opposed to intuitive ‘trial and error’) account for at least part of the lack of why scalable HaH models remain elusive globally.

In this protocol, we report a retrospective study of the planned scale-up of a HaH program in Singapore, from its pilot phase to full scale implementation. Our aim is to gain insights into barriers and facilitators influencing the scale-up process of HaH services. The primary aim is to apply well-established implementation research concepts to a HaH scale-up process to understand the barriers and drivers of the process and the associations with implementation success (or failure). Our overarching question is: How do we scale up HaH successfully?

## Methods and analysis

### The intervention, NUHS@Home

NUHS@Home is the HaH service of the National University Health System (NUHS), an academic health system in Western Singapore (12). The NUHS comprises two emergency departments, one urgent care center, three acute hospitals, three national specialist centers (cancer, cardiology and transplant), and a network of primary care clinics (including government-funded polyclinics and private general practitioner clinics as part of a primary care network).

With preliminary studies showing demand and interest from patients and providers (13, 14), NUHS@Home started was launched in September 2020 as a small-scale pilot to test the feasibility of the care model. In the pilot, multi-disciplinary care teams delivered inpatient-level care to patients at home, aligned with international HaH programs. NUHS@Home adopts an inpatient acute bed substitution approach, led by a consultant specializing in general/acute medicine. Key features of care include: (1) daily virtual ward rounds by doctors followed by home visits if physical reviews are indicated; (2) home visits by nurses and allied health professionals for procedures (e.g., intravenous medication or venipuncture) or therapy; (3) remote vital signs monitoring, and (4) a round-the-clock hotline to the care team.

Evaluation of this one-year pilot, operating with a virtual capacity of 3 beds and caring for 108 patient episodes, demonstrated that acute care at home is feasible, safe, and effective (15). From September 2021, due to the COVID-19 surge, NUHS@Home rapidly scaled to over 100 virtual beds, delivering protocolized COVID-19 care to over 2,000 patient episodes (16). Although COVID-19 service demands and patient acuity are lower and not representative of typical patients admitted to HaH programs, insights gained during this period were useful for plans for further growth.

The success of the pilot helped drive financial, regulatory, and policy shifts to support HaH scale-up nationally. The Ministry of Health launched a 2-year “sandbox” from 2022 to 2024 to support and finance these efforts. With this sandbox, patients admitted to HaH programs are eligible for subsidies similar to traditional inpatient care, with an aim to eventually transition to mainstream healthcare delivery (6). As such, NUHS@Home has planned for major expansion from 3 to 50–100 virtual beds within 3–5 years.

This protocol focuses on the evaluation of the scale-up of NUHS@Home, aiming to identify barriers and facilitators to the successful expansion of the HaH services.

### Implementation frameworks

This evaluation will combine two established implementation frameworks—the Exploration, Preparation, Implementation, Sustainment (EPIS) Framework (17, 18) and the Scale-Up Framework (19).

EPIS provides a useful macro-framework for analyzing implementation processes. It outlines four key phases—Exploration, Preparation, Implementation, and Sustainment—and identifies contextual influences across each phase. Contextual influences, defined as factors external to the intervention itself (20), may be further classified into outer context (i.e., healthcare system) and inner context (i.e., local organizational aspects). Examples of these influences include elements of leadership (within and outside the NUHS@Home team and NUHS), characteristics of the local patient population, fidelity of implementation (i.e., implementing NUHS@Home as originally designed), workforce characteristics, and more. In the field of implementation science, contextual factors have consistently been identified as key to the success or failure of implementing evidence-based interventions (21), even more than the quality and nature of supporting evidence or the complexity of the implementation process. These contextual influences are therefore the primary focus for our research and evaluation. Furthermore, EPIS offers a (chrono)logical lens through which the process of implementation can be studied as it takes place and evolves. This is useful and applicable as we study the scale-up process *in situ*, i.e., as it happens within the NUHS setting.

**Figure 1:**
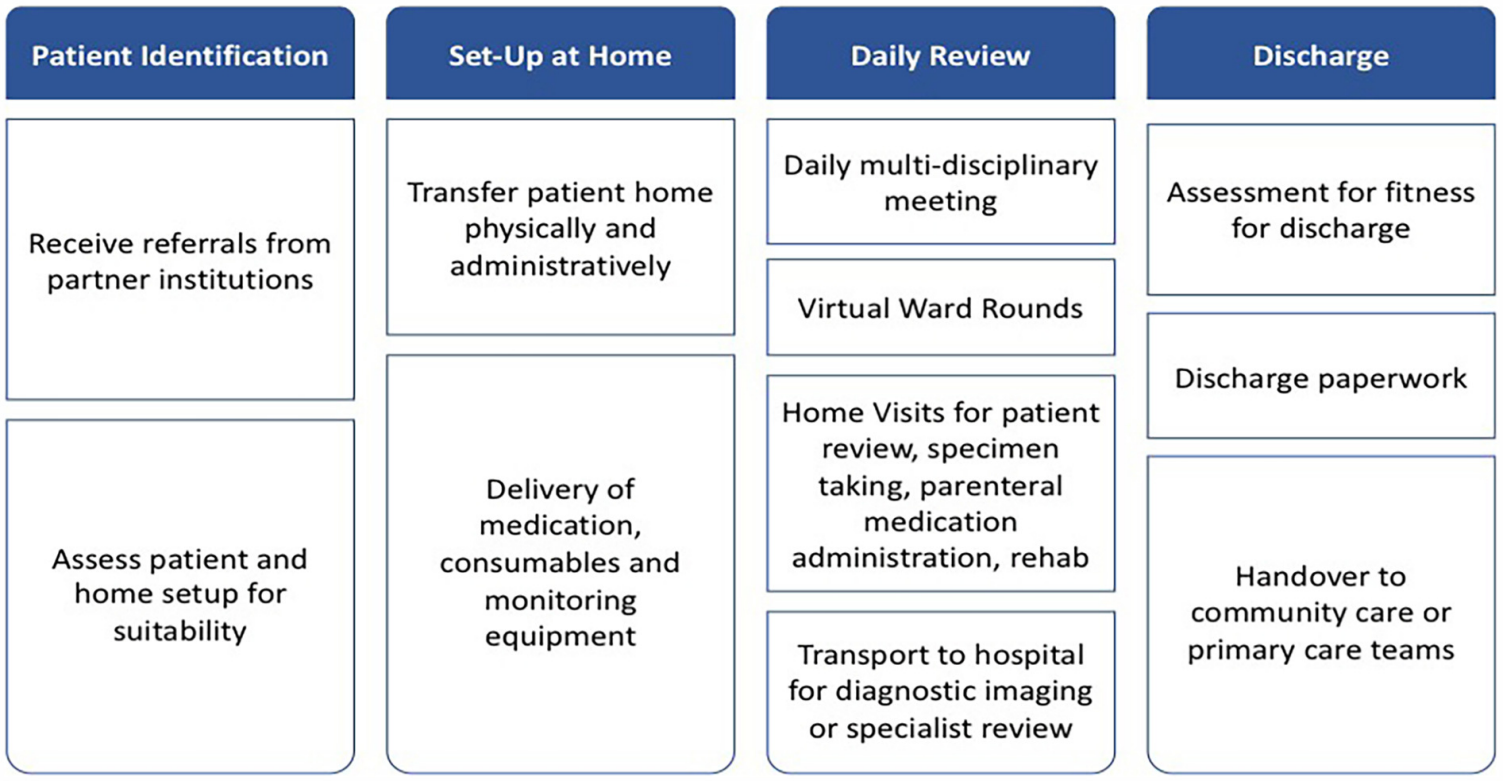
NUHS@home patient journey.

The macro approach of EPIS is complemented by an action-orientated framework to define and operationalize the specific elements of the scale-up process. This is a critically important aspect of the research, as it will lead to the ultimate ‘manualization’ of the scale-up process to create generalizable findings. The Scale-Up framework offers precisely this, in an ideal complement to EPIS. The Scale-Up framework breaks down scaling-up into 4 phases: (1) set-up, (2) develop the scalable unit, (3) test of scale-up, and (4) go to full-scale (Figure 1). Table 1 maps out the growth of NUHS@Home following these phases. The focus of our research is to study the process of developing a scalable unit (phase 2) and test of scale-up (phase 3).

**Table 1:** The scale-up framework as applied to NUHS@home.

Phases	Set-up	Develop the scalable unit	Test of scale-up	Go to full-scale
Timeline	2020–2022	2022–2024	2024–2025	2025–2030
Bed capacity	3 beds	25 beds	50–100 beds	300 beds

### Study design

This is a retrospective observational cohort study to evaluate implementation outcomes using the EPIS framework during the planned scale-up of NUHS@Home over 23 months. The primary methodology will be quantitative in approach, supported by an explanatory qualitative study with the implementation team to understand which factors are associated with the assessed implementation outcomes and how. The period of data collection will begin with the transition to phase 2 (‘develop the scalable unit’), between May 2022 to April 2024.

### Sample selection

This study will evaluate NUHS@Home, the HaH service at NUHS. NUHS@Home functions as an inpatient substitute service, replicating elements of ward-based care in the home environment. Referrals are accepted from all partner institutions in the health system, including wards, emergency departments, and primary and community care facilities. Patients are assessed for suitability based on the following criteria: (1) clinical stability, (2) ability to receive the required clinical interventions at home, (3) ability to manage toileting independently or with an available caregiver, and (4) living within the geographical catchment (Western Singapore).

The service delivery process can be summarized in four steps (Figure 1)—patient identification, set-up, daily review at home, and discharge.

Key members of the implementation team include leads from physicians, nurses, pharmacy, allied health, and operations. During the initial pilot phase, the implementation team identified 4 key priorities for scale-up: (1) development of new clinical pathways to generate demand for virtual beds, (2) recruitment and training of staff to provide inpatient-level care at home, (3) optimization of internal workflows and processes to ensure both patient safety and provider efficiency, and (4) advocacy for sustainable healthcare financing and billing. The relevant activities and EPIS contextual factors are detailed in Table 2.

**Table 2:** Priorities and activities for scale-up.

Priorities for scale up	Activities	Relevant contextual factors (EPIS)
Overall	Building an implementation team to plan and execute the following activities	Organizational characteristics (IC) Leadership (IC)
Development of new clinical pathways to generate demand for virtual beds	Working with multiple clinical stakeholders to expand clinical pathways.	Institutional leadership (OC) Patient/client characteristics (OC) Patient/client advocacy (OC)
Recruitment and training of staff to be able to provide inpatient level care in the home	Development of accreditation frameworks, careers tracks and training programs for new clinical staff	Organizational staffing (IC) Individual characteristics (IC)
Optimizing internal workflows and processes to achieve both patient safety and provider efficiency	Engaging clinical governance, medicolegal and medical informatics to develop efficient and effective work processes	Quality and fidelity of monitoring/support (IC) Infrastructure (IC) Patient/client advocacy (IC)
Advocating for sustainable healthcare financing and billing	Advocacy for healthcare policy shifts	Funding (OC) External networks (OC)

OC, outer context; IC, inner context.

### Measurements and outcomes

The primary implementation outcomes—defined as ‘the effects of deliberate and purposive actions to implement new treatments […] and are distinct from service and client (patient) outcomes’ (22)—of the scale-up process are as follows:

1. Volume: number of patient admissions to NUHS@Home and associated bed days per month
2. Operational efficiency: bed occupancy rate of NUHS@Home, defined as the number of bed days occupied/virtual bed capacity * number of days that month
3. Adoption: proportion of patients admitted to NUHS@Home/total patients admitted to affiliated hospital wards for established clinical pathways. Within the NUHS setting, these currently include: cellulitis, urinary tract infections, gastroenteritis, dengue fever, and exertional rhabdomyolysis, but may increase with the development of new clinical pathways.

Relevant clinical outcomes will include the rate of unplanned returns to hospital, 30-day readmission rate, HaH mortality rate, and rate of patient safety incidents. At the initial set-up of the NUHS@Home service, our team developed a list of patient safety indicators by reviewing inpatient hospital reporting guidelines and adapting them for NUHS@Home. These indicators include: (1) diagnosis, treatment and procedure-related complications, (2) laboratory medicine and sample-related complications, (3) peripheral venous complication-related complications, (4) medication-related complications, (5) patient falls, (6) pressure injuries, (7) sharps injury and body fluid splash related and (8) staff and visitor incidents. These indicators will be tracked throughout the study period.

In addition, basic patient demographic data (e.g., age, gender) will also be collected to provide descriptive context for the implementation analysis.

A key tenet of implementation theory is that the scale-up process and the associated outcomes of that process (summarized above) will potentially be impacted by, or at least associated with, the context of the implementation. To better understand the impact of context, we applied the EPIS framework to define contextual influences on the outcomes described above and developed a corresponding measurement plan, summarized in Table 3.

**Table 3:** Measurements of contextual influences.

Measurement (per month)	Definition
Implementation outcomes (IO)	
Volume	• Number of patient episodes • Number of bed days
Operational efficiency	• Bed days occupied • Virtual bed capacity
Adoption	• Patients admitted to NUHS@Home per clinical pathway • Total patients admitted to affiliated hospital wards for same clinical pathway
Clinical outcomes (CO)	
Clinical outcomes	• Rate of unplanned return to hospital • 30-day readmission rate • Within HaH mortality rate • Rate of patient safety issues
Outer context (OC)	
Service environment	• Bed occupancy rates of referring hospitals • Lodger volume in emergency department of referring hospitals • Number of active clinical pathways with NUHS@Home
Funding	• Source of service funding used
Institutional leadership	• Number of leadership broadcasts (and content)
External networks	• Number of engagements with external organizations
Patient/client characteristics	• Number of patients referred for each clinical pathway • % patients accepted and rejected for each clinical pathway
Patient/client advocacy	• Number of publicity material to patients and doctors
Inner context (IC)	
Organizational characteristics	• Structural changes in the organization
Leadership	• Components of leadership team
Quality and fidelity of monitoring/support	• Processes for safety review and data tracking • New policies/guidelines/clinical standard/quality indicators • Technology systems used • Volume of home visits, during and after office hours
Organizational staffing processes	• Volume of staff on service, in total, who left • Training programs for staff in existence • Referral hours (office hours, after hours, weekends)
Individual characteristics	• % staff that are permanent or rotated • Demographics, training, experience of each staff
Infrastructure	• Structure of clinical command center, pharmacy, diagnostic, lab • # of vendors, types, issues/challenges, volume of services
Patient/Client Advocacy	• Average patient satisfaction scores • % of patients who returned to hospital due to changing their mind

### Data collection and data sources

Data sources for this study will include chart review from electronic medical records, human resources data, operational data, and standardized questionnaires disseminated to the NUHS@Home Implementation Team. Collected data will be consolidated into data collection forms (DCFs) on a monthly basis (i.e., 23 data points in total) by a study team member (SL). The DCFs are available in the Supplementary Material, and the data collection strategy is summarized in Table 4.

**Table 4:** Data collection strategy.

DCF	Data	Participants	Data sources
DCF 1 (operational data)	• Indicators of volume and utilization • Outcome indicators • Patient satisfaction indicators • Average occupancy rates of referring hospitals • Average number of emergency department lodgers in referring hospitals • Volume of patients in each clinical pathway • Number of home visits conducted	Operations team	Chart review Clinical operations databases Patient satisfaction team
DCF 2 (service structure and organization)	• Source of service funding • Operating and shift hours • NUHS@Home Leadership & Administrative team	Program lead	Operations data
DCF 3 (clinical operations)	• Referral parameters for active clinical pathways • New/modifications in Clinical Guidelines	Clinical service lead	Operations data Chart review
DCF 4 (service development activities)	• Publicity Broadcasts (by hospital leadership) • Publicity Broadcasts (to staff) • Publicity Broadcasts (to patients) • Engagements with external organizations • Quality and Safety Review Process • New/modifications in Policies • Technology Systems Used • Infrastructure change(s) • External Service Vendors	Operations team to draft a response but all leads to review	Operations data
DCF 5 (staffing)	• Operating and shift hours • Clinical organizational staffing • Clinical staff • Staff demographics • Staff training	Clinical, nursing, pharmacy, allied health and operations lead to fill in separately	Operations data Human resources data

DCF, data collection form.

Color coding: implementation outcome, clinical outcome, outer context, inner context.

### Qualitative methods

We will embed qualitative methods in this study through two key components. First, a document analysis of monthly implementation team meeting minutes will be conducted retrospectively. A content analysis will be carried out by one of the study team members (SL) to identify key problems and implementation activities (administrative, management, operations, or clinical) each month. Following each meeting, the lead author (SK), who also leads the implementation team, will prospectively record any important matters not captured in the minutes or the quantitative DCFs, using rapid auto-ethnography approach (23). Our team has previously applied this method in acute medical settings (24), enabling the timely capture of contextual information and real-time adjustments to the implementation process. This ensures that research remains closely aligned with the evolving NUHS@Home program.

Second, we will conduct end-of-study focus group discussions with the core implementation team after quantitative data collection concludes. These discussions will be led by trained researchers, external to both the implementation and study teams, with expertise in qualitative methods and implementation research. They will be supervised by a senior implementation scientist (NS)—a non-clinician with over 20 years of experience in health services research in hospital settings. The focus groups will explore the following thematic areas: (1) what facilitated the scale-up process and why, (2) what did not work or hindered and why, (3) explanations of the implementation outcomes elicited in the first component of the study, and (4) contextual factors that were critical in success or failure of implementation and how precisely they affected the process. If needed, two additional implementation researchers from the NUS Centre for Behavioural and Implementation Science Interventions will be trained by NS to support the data collection. Each session is expected to last approximately 90 min and include 6–8 participants. If logistical challenges prevent group discussions, semi-structured interviews will be conducted instead.

### Sample size

Based on our pilot study, which averaged 8–10 patient episodes per month with a virtual capacity of 3 beds, we anticipate approximately 1,012 patients admitted to NUHS@Home over the 23-month study period. As this is a descriptive study with no comparison group, we have not set a minimum sample size target. If a sample size becomes necessary, we will submit an amendment to the ethics borad to extend the study period.

We expect 15–25 members of the implementation team will provide data (as outlined in Table 4) and in the focus group discussions. This will include service leads from clinical, nursing, pharmacy, allied health, and operations teams within NUHS@Home.

### Data analysis

Both implementation and clinical outcomes will be described descriptively over time to contextualize the scale-up process. We will also use statistical process control (SPC) graphs to examine variation in the three defined implementation outcomes over time. To determine the association of different contextual factors with these outcomes, each contextual factor will first be categorized as either continuous (e.g., bed occupancy rates of referring hospitals) or binary/descriptive (e.g., change in funding source). For continuous variables, unadjusted and adjusted linear regression will be used to statistically assess associations between the number of contextual factors and their values and each implementation outcome to determine whether certain contextual factors are more influential than others. For binary/descriptive variables, contextual events will be annotated on the SPC graphs to assess if a clinically significant relationship exists. These analyses will be supplemented with multivariable regression to adjust for potential confounders. This approach has been previously applied in a similar ‘naturalistic’ implementation evaluation of a hospital-based scaled intervention in the UK (25).

Qualitative data from focus group discussions and meeting minutes will be analyzed using Atlas.ti software, guided by Braun and Clarke's thematic analysis approach (26). Chat logs, transcripts, and observations will be coded inductively, i.e., without any theoretical preconceptions, and themes reflecting implementation aspects will be identified and summarized. In the first stage, data will be deconstructed systematically into first-order concepts. In the second construction stage, the concepts previously developed will be reassembled into new patterns and a constant comparison technique will be carried out to compare the incidents applicable to each emerging theme. The third and final stage of analysis will include the confirmation of the conceptualization of the new phenomenon and a descriptive narrative summary will be created using second-order concepts. The analysis will seek to establish whether the emerging theme represents a barrier or a driver. Results from the different methods will be triangulated for the final data analysis.

Interim analyses will be conducted every three months, with feedback provided to the implementation team as needed.

We anticipate that some data points may be missing due to the retrospective nature of the data collection from operational and administrative datasets. If the proportion of missing data is low (<10%) and missing at random, a complete case analysis will be conducted. If missingness is substantial or not random, we will apply appropriate methods such as multiple imputation or sensitivity analyses to assess its impact on the findings.

## Discussion

This study aims to deepen our understanding of how to effectively scale up HaH interventions, which hold significant promise in addressing critical challenges in healthcare systems. Large-scale HaH programs may reduce the demand for expanding or constructing new hospitals, curb rising healthcare costs, and shift care towards more patient-centered, home-based care models.

A key emphasis of this study is the adoption of a systematic theory-driven approach to evaluating the scale-up process, with a focus on identifying both barriers and facilitators to full-scale implementation. In doing so, the study contributes to the growing body of HaH literature, aligning with current discourse that moves beyond assessing efficacy alone and toward understanding large-scale adoption strategies.

To our knowledge, this is the first study to integrate two established implementation science frameworks—the EPIS framework and the Scale-Up framework—to analyze the scaling process of HaH interventions. This integrated approach allows for a comprehensive analysis of both the process and the contextual drivers of success or failure associated with scaling. Moreover, the study design was developed in collaboration with key implementation stakeholders from the outset, ensuring practical relevance and robust assessment of real-world conditions.

Nonetheless, there are limitations. This is an observational study treating the scale-up process as a natural experiment. As such, the evaluation is based on the service as implemented, without a control group, which restricts the ability to draw causal inferences. Consequently, the findings are limited to identifying associations rather than establishing definitive causal relationships.

Although a formal cost analysis is not included in this study, we recognize that economic viability is central to the successful scale-up and long-term sustainability of HaH services. A future follow-up economic evaluation study can be conducted to assess the cost implications and potential savings of HaH compared to conventional inpatient care, to inform healthcare financing strategies and policy decisions.

Finally, while this study protocol focuses on the evaluation of implementation and understanding contextual factors from the perspective of the implementation team, we acknowledge the importance of understanding the perspectives of referring providers. A separate qualitative study is currently underway to explore providers’ perceptions of HaH, their referral decision-making processes, and perceived barriers or facilitators to referral. Findings from that work will complement the present study and help contextualize adoption outcomes.

### Ethics and dissemination

This protocol has been approved by the National Health Group Domain Specific Review Board: Reference Number: 2023/00245. A waiver of informed consent was sought for the cohort study and document analysis of meeting minutes as individual patient data will not be collected and it involves a review of routinely collected retrospective data. For the end-of-study focus group, informed consent will be taken. The reporting of the study will follow the STROBE guidelines (27) for reporting observational studies. The results of this study will be disseminated to peer-reviewed journals, presented at conferences, and shared with policy-level stakeholders.

## Data Availability

The original contributions presented in the study are included in the article/Supplementary Material, further inquiries can be directed to the corresponding author.
