# Supplementary material for: Barriers and facilitators to scale-up of hospital-at-home: an observational cohort study protocol
Source: Front Health Serv. 2025 Jun 6;5:1571090. doi: 10.3389/frhs.2025.1571090 (PMC12179168; doi:10.3389/frhs.2025.1571090)
Supplement: Supplementary file 1 [file Datasheet1.pdf]

Supplementary Material
Data Collection form 1: Operational Data
Month: _____

1. **Indicators of volume and utilisation**

Indicator	Value
1. Number of patient episodes	
2. Number of virtual ward bed days	
3. Maximum bed capacity	

2. **Outcome indicators**

Indicator	Value
1. Number of unplanned return to hospital	
2. Number of 30 day readmissions	
3. Number of mortality associated to episode	
4. Patient safety issues (incident reports)	

3. **Patient satisfaction indicators**

Patient Satisfaction Scores	Value
Number of patients who responded	
Average patient satisfaction score	
% that would choose this again	

4. **Average occupancy rates of referring hospitals**

	Average Bed Occupancy Rate	Average ED Lodger Numbers
National University Hospital		
Alexandra Hospital		
Ng Teng Fong General Hospital		

5. **Volume of patients in each clinical pathway**

	Number of patients admitted to NUHS@Home	Number of virtual ward bed days	Number of patients admitted to NUH	Number of patients admitted to NTFGH	Number of patients admitted to AH
**e.g. cellulitis**					

6. **Number of home visits conducted**

	Office Hours	After Office Hours
**NUHS@Home doctors**		
**NUHS@Home nurse**		
**NUHS@Home allied health professionals**		
**Vendor nurse**		

Data Collection Form 2: Service Structure and Organisation

1. **Source of service funding**

   Any changes in the last month? ¨ Yes ¨ No

   If yes, what was the change?

2. **Operating and shift hours**

   Any changes in the last month? ¨ Yes ¨ No

   If yes, what was the change?

3. **NUHS@Home Leadership & Administrative team**

   Any changes in the last month? ¨ Yes ¨ No

   If yes, what was the change?

Data Collection Form 3: Clinical Operations

Month: _______

1. **Referral parameters for active clinical pathways***

	**Existing/new**	**Number of referrals**	**Number not accepted as patient unsuitable**	**Number not accepted as patient declined**
**e.g. cellulitis**				

*1st column should tally with DCF1 question 5.

2. **List New Clinical Guidelines/Protocols (if any)**

	**Title**
1	e.g. hypoglycaemia protocol
2	
3	

Data Collection Form 4: Service development activities

Month: _______

1. **Publicity Broadcasts**

By Whom	Number this month
Hospital Leadership to all staff	
Department leads to department staff	
Hospital to patients (including media)	

2. **Engagements with external organisations**

Category	Number this month
Ministry of Health	
Existing or prospective hospital-at-home programmes	
Prospective external vendors	
Potential partner organisations (e.g. SLH)	

3. **Policies / Quality and Safety Review Process**

Category	Number this month
New quality and safety processes	
New policies	

4. **Technology Systems Used**

New technology systems deployed	Number this month
Patient monitoring	
Patient communications	
Clinical communications	
Others	

5. **Infrastructural changes**

Infrastructure	Change (yes/no)
Clinical Command Centre	
Pharmacy	
Diagnostic Imaging	
Laboratory	
<insert new>	

6. **External Service Vendors**

Service delivered	Vendor Name	Volume of service
e.g. phlebotomy, IV infusion, courier services, ambulance (1 service per row)		

Data Collection Form 5: Staffing

Month: _______

Job Group: Physician / Nursing / Pharmacy / Allied Health / Coordinators

1. **Operating and shift hours**

   Any changes in the last month? ¨ Yes ¨ No

   If yes, what was the change?

2. **Clinical organisational staffing**

   Any changes in the last month? ¨ Yes ¨ No

   If yes, what was the change?

3. **Clinical staff**

Staff	Number
Total staff	
New staff this month	
Staff who left this month	

4. **List Staff Training Activities (if any)**

	Title
1	e.g. POCT training
2	
3	
